# Comparative Evaluation of Conventional Therapy With and Without Use of Diode Laser (DL) in the Treatment of Chronic Generalized Periodontitis: A Clinico-Microbiological Study

**DOI:** 10.7759/cureus.35720

**Published:** 2023-03-03

**Authors:** Ankita Saha, Pallavi Kamble, Sachin B Mangalekar

**Affiliations:** 1 Periodontology, Bharati Vidyapeeth (Deemed to be University) Dental College and Hospital, Sangli, IND

**Keywords:** clinical therapy, periodontal therapy, periodontitis, flap surgery, diode laser

## Abstract

Introduction:Chronic periodontitis is caused by a persistent and expanding interaction between a subgingival pathogenic microbial biofilm and the host immune system. The host's reaction to local factors directly influences the inflammation and bone loss that result from these interactions. Depending on variables like the severity of soft tissue damage and bone loss, treatment options can range from nonsurgical to surgical. Nonsurgical treatments are frequently used as the first-line therapy for inflammatory periodontal disease. In fact, careful scaling and root planing (SRP), a nonsurgical treatment, has been extensively studied and shown to be a highly predictable and effective therapy. According to recent research, using a diode laser (DL) in addition to standard SRP may reduce bacterial count and reinfection significantly. Laser therapy could be helpful in treating periodontal disease because of its antibacterial and detoxifying effects. The goal of this study is to investigate whether using a DL in addition to conventional flap surgery enhances patient outcomes for those with chronic generalized periodontitis.

Materials and methods: The 12 participants in this split-mouth trial with chronic generalized periodontitis were the main subject of the study. All of them had probing pocket depths (PPDs) of at least 5 mm after the initial phase of treatment. Each patient in the control group (Group A) and test group (Group B) received a conventional flap after being randomly assigned to one of the groups. Group B underwent a conventional flap with a 980 nm DL, whereas those in Group A did not receive any DL therapy. Periodontal pockets in both groups were evaluated at baseline, 45 days, and 90 days after a sub-gingival plaque test. Quantitative real-time polymerase chain reactions were used to examine the presence of red complex organisms in the plaque sample.

Results: From baseline to 45 days and then to 90 days, clinical attachment loss (CAL), plaque index (PI), and gingival index (GI) all significantly decreased. However, results from 45 days to 90 days were statistically non-significant, with the exception of the GI, where Group B results were significantly different from Group A results from 45 days to 90 days. On the other hand, when a DL was combined with conventional flap surgery in the test group, the quantity of red complex bacteria was significantly decreased.

Conclusion:When DL was used in conjunction with conventional flap surgery, the results showed that CAL, PI, and GI were all significantly reduced while the quantity of red complex bacteria was also significantly decreased.

## Introduction

Infected gingiva and supporting tissues lead to a condition known as periodontal disease, characterized by chronic inflammation. A review of the literature reveals that this "infectious disease" is characterized by inflammation within the oral supporting tissues, increasing attachment loss, and bone loss. Microbial plaque and calculus are major contributors to this complex illness. Bacteria of several types may colonize and form a biofilm inside the tooth plaque as it continues to build up. Subgingival plaque is home to a diverse microbiota, although gram-negative anaerobic bacteria make up the bulk of the population. Aggregatibacter is one of the most often found microbes at elevated concentrations in periodontal lesions [1]. Loss of alveolar bone and attachment loss are the peculiar features of periodontal inflammation [2]. There is a larger inflammatory content and a latent virus in plaque-induced gingivitis, both of which become active in response to factors such as immunosuppression, infection, stress, and hormonal impact, ultimately inducing the periodontopathogenic characteristic and resulting in periodontal damage [3]. Sites infected with the herpes virus are more likely to break down, and the presence of the virus improves bacterial adhesion, leading to an increase in bacterial load that, in turn, increases and modifies inflammatory mediators [4]. Herpes was linked to less aggressive behavior than chronic diseases [5]. Chronic periodontitis is immune-mediated; therefore, each person's experience with it is unique in terms of its start, development, and severity. Patients may also exhibit changes in their peripheral monocytes, which are associated with either reduced lymphocyte reactivity or an amplified B-cell response. Pro-inflammatory mediators are produced by multiple cell types in the periodontium, including macrophages, periodontal ligament cells, gingival fibroblasts, and epithelial cells, and they modify both innate and adaptive immune responses. There are no true immunological distinctions between the two disease entities; rather, changes in susceptibility to illness reflect variations in disease intensity [6].

It is the persistent and growing interaction between a subgingival pathogenic microbial biofilm and the human immune system that leads to chronic periodontitis. The inflammation and bone loss that result from these interactions are directly related to the teeth. Treatment options range from nonsurgical to surgical based on factors such as the extent of soft tissue injury and bone loss. Nonsurgical treatments are frequently used as first-line therapy for inflammatory periodontal disease. As a matter of fact, nonsurgical treatment comprising careful scaling and root planing (SRP) has been widely studied and demonstrated to be a highly predictable and effective therapy [1]. Current theories of periodontitis account for the role played by microbes, inflammatory host responses, and environmental variables [7,8]. The damage they do to tissues, both directly and via the host's immune response, plays a significant part in the onset of disease. SRP is a treatment whose primary goal is to restore gingival health by eliminating the irritants that have been causing it (i.e., plaque, calculus, necrotic cementum, and endotoxins inserted on the root surface). Problems that may arise during SRP include poor access in subgingival site and to the tooth regions such as furcation sites as well as concavities present on the root surface, which can affect the instrumentation success. [8].

The ultimate goals of periodontal therapy for inflammatory periodontal disease are the elimination of these causes, the restoration of function, and the regeneration of lost supporting tissues. It is impossible for conventional nonsurgical mechanical therapy to eradicate all bacterial deposits and related toxins from the root surface due to inaccessible areas such as furcations, concavities, and forming grooves [9]. Therefore, deeper pockets are a sign that flap surgery is necessary, leading to a higher rate of rapid decrease in pockets and attachment gain [10]. Traditionally, periodontal disease has been treated with a combination of traditional mechanical debridement and surgical periodontal procedures. Improved hemostasis, sterilization of the incision or target surface area, and decreased post-treatment tissue edema and swelling are just some of the ways in which diode laser (DL) outperforms more traditional surgical methods [11]. DLs may be utilized for soft-tissue operations such as gingival cutting and coagulation, soft-tissue curettage, and sulcular debridement without causing damage to the surrounding dental hard tissues [12]. It has been shown that bacteria and inflammation may be drastically reduced by employing a DL in conjunction with traditional SRP [2]. Laser therapy is helpful in periodontal therapy because it has good tissue penetration and is well absorbed in pigmented tissues, it can specifically target the pigmented bacteria and granulation tissue. The literature presents conflicting reports of the clinical and microbiological effects of laser treatment as an adjunct to SRP. As a result, this study aims to learn whether DL therapy can be utilized as an adjunct to conventional flap surgery in the treatment of chronic periodontitis [11].

## Materials and methods

Study protocol

The institutional review board of Bharati Vidyapeeth (Deemed to be University) Dental College and Hospital, Sangli, conferred ethical approval for this research with reference number BV(DU)MC&H/IEC/2019-20/D-20. By selecting two different mouth quadrants from each of our 12 participants, we used a split-mouth design. Patients were assigned at random to either the control group (Group A), where they received conventional flap treatment, or the test group (Group B), where conventional flap treatment was performed on randomly chosen quadrants while using a 980 nm DL. Patients were than the age of 18, have six or more teeth in each quadrant, and have a pocket probing depth (PPD) of at least 5 mm following Phase-I therapy. Patients were ineligible if they smoked, had a systemic infection, had undergone periodontal surgery on the affected area within the previous six months, used antibiotics or steroids within the previous three months, or had undergone pregnancy.

Methods

12 people with chronic generalized periodontitis were the main subjects of this split-mouth study. All of their evaluation pocket depths (PDs) were 5 mm or more following Phase-I treatment. Groups A and B were randomly assigned to each patient's two ipsilateral (same-side) quadrants using a coin toss. Group A's patients received conventional flap therapy. At the test site, a DL was used in addition to conventional flap surgery. Samples of subgingival plaque were taken from both groups at the start of the review, after 45 days, and once more after 90 days. We searched for the presence of red complex organisms using quantitative real-time polymerase chain reactions in plaque samples.

**Figure 1:**
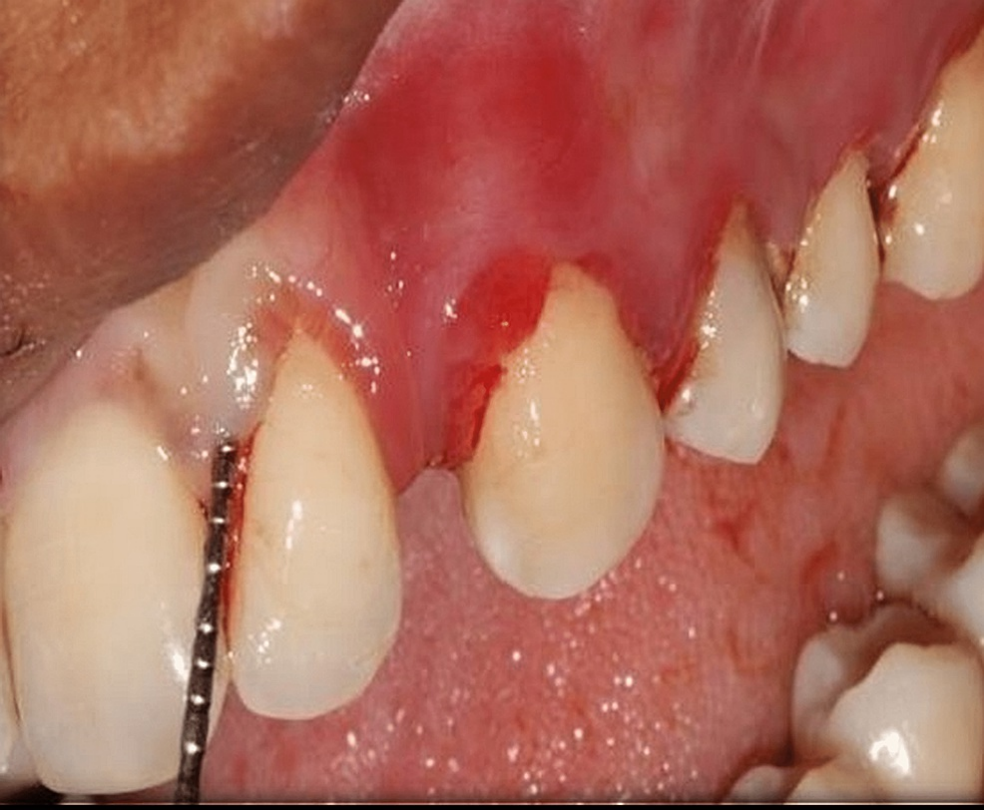
Preoperative probing

Patients with untreated chronic periodontitis were selected if at least two of their four periodontal pockets had probing depths of 5 mm or higher. Group A received conventional flap surgery, while Group B received conventional flap surgery combined with a 980 nm DL. Selected quadrants were divided into two groups. All participants' oral health was assessed using the following criteria:

*Clinical benchmarks*: The duration of baseline measurements of the clinical attachment level, pocket probing depth (PPD), gingival index (GI), and plaque index (PI) was compared to the results of periodontal treatment (PT). Each tooth's four points were measured using a University of North Carolina (UNC) 15 probe by the same researcher (Figure 1). Prior to enrollment, participants gave informed written consent.

*Microbiological parameters*: Subgingival plaque at the deepest periodontal site was sampled from the Groups A and B prior to treatment. Plaque samples were taken as soon as possible, put in a transport medium, and sent for a polymerase chain reaction analysis of red complex bacteria. Prior to surgery, the operator performed a Phase-I treatment, which entails SRP of the entire mouth and offering oral hygiene advice.

**Figure 2:**
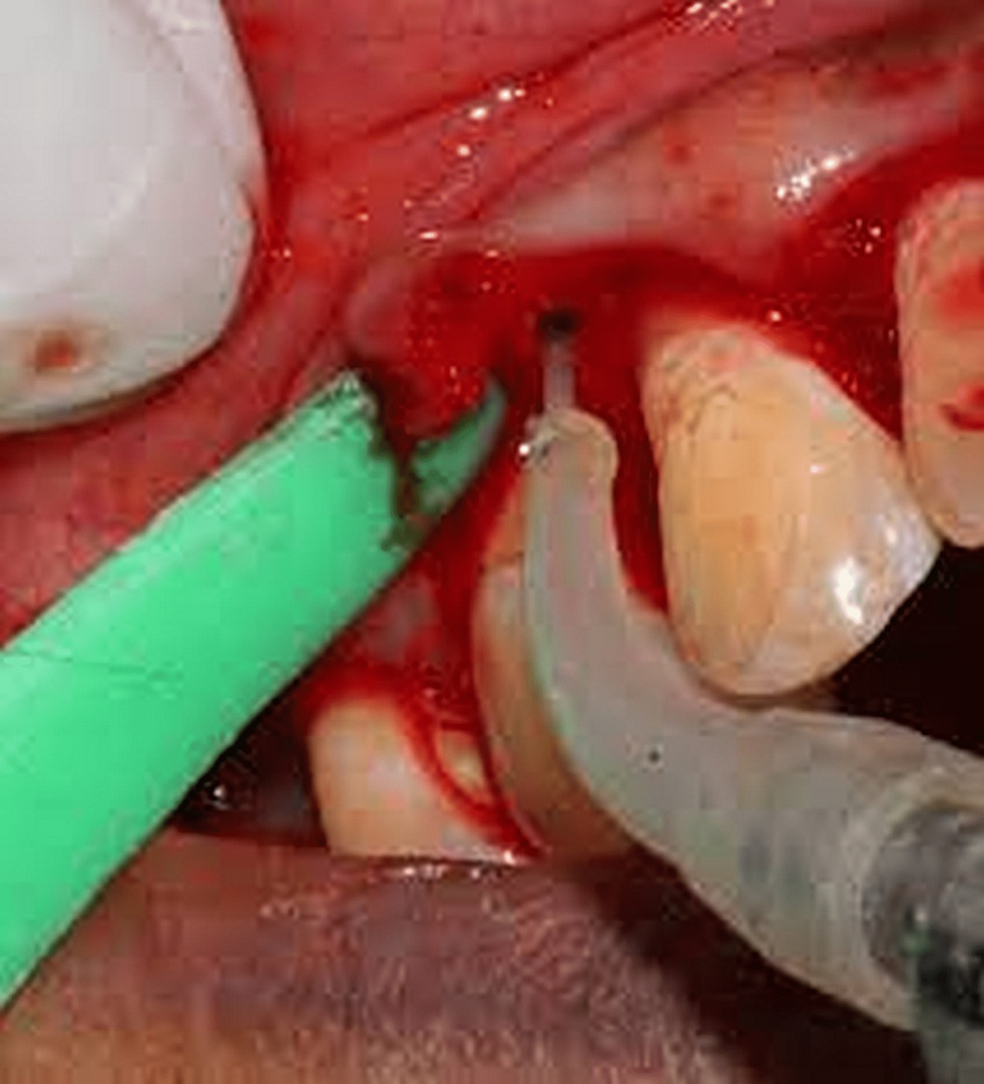
Laser application as an adjunct to flap surgery

Four weeks after completing Phase-I treatment, the same surgeon performed surgeries on every patient in both groups. In Group B, the surgical site was properly isolated before being anesthetized with 2% lidocaine hydrochloride and adrenaline (1:200,000). Each individual tooth segment or affected area was prepared by making crevicular incisions in the gingiva and the alveolar bone with a Bard-Parker No. 12 blade. A mucoperiosteal flap was reflected, the granulation tissue was debrided, and root planning was carried out using a periosteal elevator. A 980 nm DL unit with a 7 mm long, 400-micron diameter fiber-optic tip was used in Group B. The device could produce a maximum of 2 W of power. In order to avoid the underlying bone and teeth when operating in contact mode, the fiber-optic tip of the laser was angled at 45 degrees away from the inner surface of the flap (Figure 2).

The inner lining of the flap and palatal flaps were treated with horizontal overlapping strokes for approximately 10 seconds per tooth. The area was then irrigated using regular saline. The burned layer that had formed on the pocket wall was removed using a high-volume suction device as well as moist gauze. After that, the area was watered with regular saline, the field was cleaned up with high-volume suction equipment and the flap was stitched back together. On the control side, conventional flap surgery with mechanical debridement was performed, and the initial healing of the wound was completed using (3-0) black silk.

**Figure 3:**
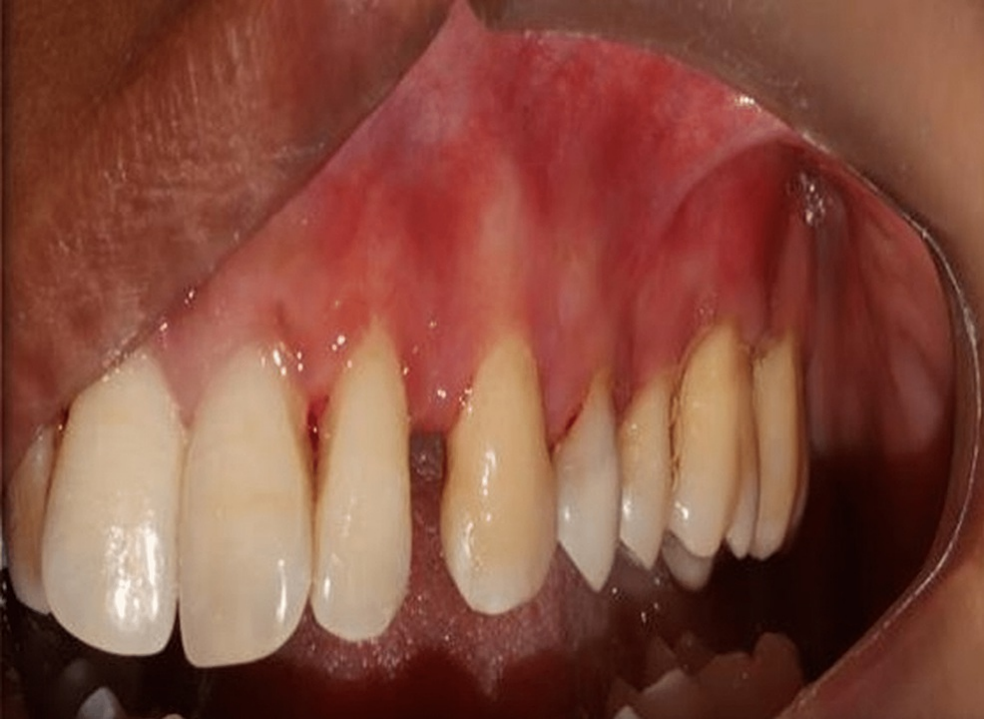
Postoperative image after 90 days

*Postoperative care and monitoring*: Patients were advised to take specific actions after surgery. An antibiotic (amoxicillin 500 mg three times daily for five days) and painkillers were used in the course of treatment (aceclofenac and paracetamol twice a day for three days). For a period of seven days, patients were told to gargle with a 0.2% chlorhexidine solution after every meal. Everyone was placed on a maintenance protocol following surgery. Recall visits in this instance were scheduled for the 45th and 90th days (Figure 3).

At every subsequent appointment, oral hygiene was examined and emphasized. The periodontal boundaries were reassessed and subgingival plaque was tested in both groups after 45 and 90 days. The traditional polymerase chain reaction was used to analyze plaque tests for the presence of Porphyromonas gingivalis (Pg), Treponema denticola (Td), and Tannerella forsythia (Tf), three red complex organisms.

*Statistical analysis*: The study made use of a statistical significance test. Using descriptive statistics, averages, dispersion, and frequency distributions were examined. The data sets were compared using an unpaired t-test to identify significant variations between the categories. To make comparing data across time periods easier, an analysis of variance (ANOVA) was carried out. It was determined that a p value of 0.05 or less and a highly significant value of p = 0.001 were required for statistical significance.

## Results

Two men (16.7%) and 10 women (83.3%) made up Group A and Group B. In a Chi-square test for comparison, there was no discernible change (p>0.05) (Table 1).

**Table 1:** Gender comparison between Group A (control group) and Group B (test group) NS: Not significant

	Group A (Control group)	Group B (Test Group)	Chi square test value	P value, Significance
Male	2 (16.7%)	2 (16.7%)	0.000	1.000 (NS)
Female	10 (83.3%)	10 (83.3%)		

Overall, a highly statistically significant difference (p=0.001) was seen in Group A with regards to the change in mean PI from baseline 2.94+0.36 to 2.09+0.32 at 45 days and to 1.63+0.31 at 90 days. It was determined that the statistically significant change (decrease) occurred only in the mean PI between the days 45 and 90. Group B showed a statistically significant drop in PI from baseline 2.93 +0.34 to 1.71 +0.44 at 45 days and then to 1.18+0.36 at 90 days. It was determined that the statistically significant change (decrease) occurred only in the mean PI between the days 45 and 90 (Table 2).

**Table 2:** Intragroup comparison of mean PI score between Group A (control group) and Group B (test group) ANOVA: Analysis of variance; PI: Plaque index; SD: Standard deviation

PI	Group A (Control Group) Mean (SD)	Group B (Test Group) Mean (SD)
Baseline	2.94±0.36	2.93±0.34
45 Days	2.09±0.32	1.71±0.44
90 Days	1.63±0.4	1.18±0.37
One-way ANOVA F test value	F=38.709	F=63.378
p value, Significance	p<0.001	p<0.001
Baseline vs 45 days	p<0.001	p<0.001
Baseline vs 90 days	p<0.001	p<0.001
45 days vs 90 days	p=0.013	p=0.006

At baseline, the mean GI in Group A was 2.56+0.27, but after 45 days, it had reduced to 1.93+0.4, and after another 45 days, it had reduced to 1.5 at 90 days, representing an overall and pairwise highly significant difference (p=0.001) in relation to change (decrease). The only statistically significant variation was a decrease in the mean GI from 45 days to 90 days (p<0.05). The mean GI in Group B decreased from 2.7+0.32 at baseline to 1.42+0.26 after 45 days and further decreased to 0.9+0.03 after 90 days, with these changes being highly statistically significant (p<0.001) (Table 3).

**Table 3:** Intragroup comparison of GI between Group A (control group) and Group B (test group) ANOVA: Analysis of variance; GI: Gingival index; SD: Standard deviation

GI	Group A (Control Group) Mean (SD)	Group B (Test Group) Mean (SD)
Baseline	2.56±0.27	2.7±0.32
45 Days	1.93±0.4	1.42±0.26
90 Days	1.5±0.3	0.9±0.13
One-way ANOVA F test value	F=30.538	F=157.373
p-value, Significance	p<0.001	p<0.001
Baseline vs 45 days	p<0.001	p<0.001
Baseline vs 90 days	p<0.001	p<0.001
45 days vs 90 days	p=0.01	p<0.001

After baseline, the mean PD in Group A was 5.71 +0.56 at baseline, at 45 days it had dropped to 3.41 +0.45 and at 90 days it had fallen to 2.98+0.12. These changes, both individually and in comparison to one another, were statistically significant (p<0.001). It was determined that the decrease in mean PD from 45 days to 90 days was not statistically significant (p>0.05). Change (reduction) occurred in mean scores from baseline value of 5.53+0.58 to 2.78+0.46 after 45 days and then to 2.57+0.29 after 90 days in Group B, which was statistically significant (p<0.001) (Table 4).

**Table 4:** Intragroup comparison of mean PD between Group A (control group) and Group B (test group) ANOVA: Analysis of variance; PD: Pocket depth; SD: Standard deviation

PD	Group A (Control Group) Mean (SD)	Group B (Test Group) Mean (SD)
Baseline	5.71±0.56	5.53±0.58
45 Days	3.41±0.45	2.78±0.46
90 Days	2.98±0.43	2.57±0.32
One-way ANOVA F test value	F=109.896	F=146.91
p value, Significance	p<0.001	p<0.001
Baseline vs 45 days	p<0.001	p<0.001
Baseline vs 90 days	p<0.001	p<0.001
45 days vs 90 days	p=0.089	p=0.520

Clinical attachment loss (CAL) was compared between Group A and Group B. An overall and pairwise highly statistically significant difference (p=0.001) was detected in Group A in regard to change (decline) in mean score from 5.85+0.67 at baseline to 3.48+0.45 after 45 days, and further fall to 2.97+0.53 after 90 days (0.39). The only statistically non-significant change (decrease) was seen in the mean CAL score from 45 days to 90 days (p>0.05). Mean CAL scores in Group B decreased from 5.55+0.58 at baseline to 2.78+0.46 after 45 days and 2.57+0.46 after 90 days, with both changes being highly statistically significant (p0.001) and (0.32). The only significant change (decrease) in mean measures was observed to be from 45 days to 90 days, however, this did not reach statistical significance (p>0.05) (Table 5).

**Table 5:** Intragroup comparison of mean CAL between Group A (control group) and Group B (test group) ANOVA: Analysis of variance; CAL: Clinical attachment loss; SD: Standard deviation

CAL	Group A (Control Group) Mean (SD)	Group B (Test Group) Mean (SD)
Baseline	5.85±0.67	5.55±0.58
45 Days	3.48±0.45	2.78±0.46
90 Days	2.97±0.39	2.57±0.32
One-way ANOVA F test value	F=104.896	F=146.91
p value, Significance	p<0.001	p<0.001
Baseline vs 45 days	p<0.001	p<0.001
Baseline vs 90 days	p<0.001	p<0.001
45 days vs 90 days	p=0.059	p=0.517

In order to determine statistical significance, as red complex organisms are associated with gingival bleeding and inflammation, we compared Group A and Group B in terms of their mean Pg counts. It was found that there was a significant difference in between the two groups with regard to Pg production. For this study, we compared the usual amount of Td in the two groups. Td counts rose more rapidly in Group B than in Group A from baseline to 45 days, while the difference was not statistically significant (p > 0.05). The mean Td count increased dramatically (p<0.05) from 45 days to 90 days. In this study, we compared the usual amount of Tf in the two groups. Tf levels between Groups A and B were not drastically different (p>0.05).

## Discussion

The end goals of periodontal therapy are the elimination of etiological factors, restoring function, and regeneration of the supporting tissue lost owing to inflammatory periodontal disease. As a result of the difficulty in reaching the root surface in areas like furcations, concavities, and developing grooves, conventional nonsurgical mechanical treatment is unable to eradicate all bacterial deposits and associated toxins [6]. As a consequence, deeper pockets require flap surgery, with the subsequent benefits of pocket reduction and attachment gain [10]. More and more often, lasers are being used in periodontics as an adjunct to traditional surgical procedures because of the potential benefits they provide. New connective tissue attachment happens with the use of lasers and prevent epithelium ingress from the marginal gingiva and allows creation of new junctional epithelial attachment [13].

Clinical variables were assessed at 45 and 90 days in the current investigation. Red complex bacteria such (as Pg, Td, and Tf) were analyzed by microbiologically quantitative conventional PCR analysis at baseline, 45 days, and 90 days. Since dental plaque was identified as the causative factor in periodontal disease, preventing the buildup of this biofilm on the teeth has been the primary focus of periodontal therapy. During PT, it is crucial to evaluate the plaque level. In this investigation, we thus kept track of plaque presence or absence. In the current investigation, the plaque was measured using the PI at baseline, 45 days, and 90 days [14,15].

The PI of the two groups differed significantly from the start of the study to the end of the 90-day follow-up period. Although authors demonstrated a considerable improvement after therapy, they also revealed a minor improving trend over time. Patients were invited back into the trial after 45 and 90 days to receive oral reinforcement, which may have contributed to an even greater decline in PI score. After 90 days of follow-up, both groups showed statistically significant increase in GI compared to the start of the trial. Both Groups A and B exhibited a substantial reduction in GI from baseline to three months. The difference in PPD improvement between Groups A and B was large, but the difference was not statistically significant (p>0.05). Studies by Khan et al. produce similar findings [16]. Researchers demonstrated that the laser-treated group fared better than Group A. It was discovered that Group B had a higher overall mean CAL score from baseline to 90 days than Group A, although this difference did not reach statistical significance (p>0.05).

These findings corroborated those of Angelov et al. [17]. Following DL-assisted Kirkland flap surgery, new connective tissue attachment was established with a gain in CAL, and 980 nm DL was employed for more complete debridement. When the capillaries and lymphatics are sealed off by a laser, no chemical mediators can be released to stimulate epithelial migration. Both groups saw a reduction in the number of microorganisms they were exposed to. While Assaf et al. found same findings as in this study, Euzebio et al. found that standard periodontal therapy treatments were not shown to have any further advantages based on clinical and microbiological data using high-intensity DLs [18,19]. Based on the collected data, it was shown that adding DL to traditional flap surgery significantly decreased PI, bleeding on probing (BOP), and PPD while increasing CAL. In addition, as compared to Group A, there was a significant decrease in the number of red complex bacteria Pg, Td, and Tf. As combined with traditional flap surgery, DL considerably decreased PI, BOP, and PPD while increasing CAL. Additionally, the amount of red complex bacteria Pg, Td, and Tf was significantly reduced when compared to the Group A.

The study limitations were that the sample size was very small; very little number of periodontal bacteria were studied and more bacteria need to be evaluated.

## Conclusions

Non-surgical periodontal therapies like SRP have demonstrated predictable and effective therapy. However, there have also been some failures, such as inadequate visibility and accessibility in deep pockets, which termed for surgical intervention to help with the etiologic factor's removal and the restoration and regeneration of the lost support tissue. Clinical outcomes have improved when lasers have been used as an adjunct to conventional flap surgery. The study came to the conclusion that using lasers in addition to flap surgery can produce outcomes that are more promising than those of conventional treatments. The long-term effects of using a DL during flap surgery must therefore be evaluated through additional longitudinal studies with a larger sample size.

## References

[1] Gokhale SR, Padhye AM, Byakod G, Jain SA, Padbidri V, Shivaswamy S (2012). A comparative evaluation of the efficacy of diode laser as an adjunct to mechanical debridement versus conventional mechanical debridement in periodontal flap surgery: a clinical and microbiological study. Photomed Laser Surg.

[2] Slots J (2010). Herpesviral-bacterial interactions in periodontal diseases. Periodontol 2000.

[3] Taha M, Al-Rassam ZT (2014). Chronic periodontitis and herpes viruses. J Hum Virol Retrovirol.

[4] Bilichodmath S, Mangalekar SB, Sharma DC (2009). Herpesviruses in chronic and aggressive periodontitis patients in an Indian population. J Oral Sci.

[5] Ford PJ, Gamonal J, Seymour GJ (2010). Immunological differences and similarities between chronic periodontitis and aggressive periodontitis. Periodontol 2000.

[6] Kornman KS, Page RC, Tonetti MS (1997). The host response to the microbial challenge in periodontitis: assembling the players. Periodontol 2000.

[7] Kornman KS (2008). Mapping the pathogenesis of periodontitis: a new look. J Periodontol.

[8] Adriaens PA, Edwards CA, De Boever JA, Loesche WJ (1988). Ultrastructural observations on bacterial invasion in cementum and radicular dentin of periodontally diseased human teeth. J Periodontol.

[9] Aoki A, Sasaki KM, Watanabe H, Ishikawa I (2004). Lasers in nonsurgical periodontal therapy. Periodontol 2000.

[10] Heitz-Mayfield LJ, Lang NP (2013). Surgical and nonsurgical periodontal therapy. Learned and unlearned concepts. Periodontol 2000.

[11] Moritz A, Schoop U, Goharkhay K, Schauer P, Doertbudak O, Wernisch J, Sperr W (1998). Treatment of periodontal pockets with a diode laser. Lasers Surg Med.

[12] Fisher SE, Frame JW, Browne RM, Tranter RM (1983). A comparative histological study of wound healing following CO2 laser and conventional surgical excision of canine buccal mucosa. Arch Oral Biol.

[13] Kreisler M, Al Haj H, Daubländer M (2002). Effect of diode laser irradiation on root surfaces in-vitro. J Clin Laser Med Surg.

[14] loe H (1967). The gingival index, the plaque index and the retention index systems. J Periodontol.

[15] Silness J, Loe H (1964). Periodontal disease in pregnancy. II. Correlation between oral hygiene and periodontal condition. Acta Odontol Scand.

[16] Khan F, Chopra R, Sharma N, Agrawal E, Achom M, Sharma P (2021). Comparative evaluation of the efficacy of diode laser as an adjunct to modified Widman flap surgery for the treatment of chronic periodontitis: a randomized split-mouth clinical trial. J Indian Soc Periodontol.

[17] Angelov N, Pesevska S, Nakova M (2009). Periodontal treatment with a low-level diode laser: clinical findings. Gen Dent.

[18] Assaf M, Yilmaz S, Kuru B, Ipci SD, Noyun U, Kadir T (2007). Effect of the diode laser on bacteremia associated with dental ultrasonic scaling: a clinical and microbiological study. Photomed Laser Surg.

[19] Euzebio VT, de Andrade AK, Toaliar JM (2013). Clinical and microbiological evaluation of high intensity diode laser adjutant to non-surgical periodontal treatment: a 6-month clinical trial. Clin Oral Investig.

